# Deciding when to stop: efficient experimentation to learn to predict drug-target interactions

**DOI:** 10.1186/s12859-015-0650-9

**Published:** 2015-07-09

**Authors:** Maja Temerinac-Ott, Armaghan W Naik, Robert F Murphy

**Affiliations:** 1 Freiburg Institute for Advanced Studies, University of Freiburg, Freiburg, Germany; 2 Computational Biology Department, Carnegie Mellon University, 5000 Forbes Ave, Pittsburgh, 15213 PA USA; 3 Departments of Biological Sciences, Biomedical Engineering and Machine Learning, Carnegie Mellon University, 5000 Forbes Ave15213, Pittsburgh, PA USA

**Keywords:** Active learning, Drug-target prediction, Simulation, Matrix factorization, Regression

## Abstract

**Background:**

Active learning is a powerful tool for guiding an experimentation process. Instead of doing all possible experiments in a given domain, active learning can be used to pick the experiments that will add the most knowledge to the current model. Especially, for drug discovery and development, active learning has been shown to reduce the number of experiments needed to obtain high-confidence predictions. However, in practice, it is crucial to have a method to evaluate the quality of the current predictions and decide when to stop the experimentation process. Only by applying reliable stopping criteria to active learning can time and costs in the experimental process actually be saved.

**Results:**

We compute active learning traces on simulated drug-target matrices in order to determine a regression model for the accuracy of the active learner. By analyzing the performance of the regression model on simulated data, we design stopping criteria for previously unseen experimental matrices. We demonstrate on four previously characterized drug effect data sets that applying the stopping criteria can result in upto 40 *%* savings of the total experiments for highly accurate predictions.

**Conclusions:**

We show that active learning accuracy can be predicted using simulated data and results in substantial savings in the number of experiments required to make accurate drug-target predictions.

## Background

A critical step in developing new therapeutics is frequently to conduct large scale searches for potential drugs that can affect a desired target. Recently, it has become clear that finding successful drugs also requires searching for the absence of undesired effects on other targets. This need can often not be met by exhaustive experimentation due to cost, but selective experimentation driven by machine learning (a process referred to as *active learning*) may provide an alternative [1]. The heart of active learning is having good predictive models to guide experimentation. Recent studies show that drug-target prediction algorithms can speed-up the discovery of new drugs (e.g., [2–5]).

Current drug-target prediction methods are coarse grained over at most a handful of ’campaigns’. In these, a classifier is trained with relatively large amounts of training data resulting from exhaustive screening, and then verified on a small test set. These data are generally identified manually, and limited to human ’expert’ knowledge. This process is generally only performed once, or at most a handful of times due to the expense of exhaustive screening over many compounds. This procedure limits the generalization capability of the model and does not allow for an optimal exploration of the drug-target interaction space. Alternatively, active learning methods can be used to iteratively build a model of drug-target interactions. Instead of relying on large training data sets, the active learning procedure enlarges the training set stepwise, guided by the predictions on small, automatically-selected test sets. Thus time and experimental costs are spent on improving the general model rather than for the verification of a small specific model that does not account for the large space of chemical compounds and targets. The general model has the potential to predict side-effects early on in the drug design process, since a larger number of drugs and targets are considered in the drug-target prediction matrix. A critical point when using active learning to guide experimentation is to decide when to stop, since the goal is to perform as few experiments as possible in order to have the best model. The best stopping time is reached when adding new experiments to the training set will not appreciably improve the accuracy on the test set. The difficulty, of course, is that calculating the true accuracy of the model requires all of the data. Therefore, reliable methods for predicting the accuracy of the current model during an active learning cycle are desired. This would allow experimentation to stop when a predefined confidence on the output of the model is reached.

A natural question is how such an active learning strategy is related to classical statistical approaches [6, 7] to design experiments with incomplete coverage of factors to estimate response surfaces. In the case of a large number of parameters in the model (multiple drugs and multiple targets), these methods are very slow and adapting them to model a large number of parameters is challenging [8]. Furthermore, the most critical difference between the active learning strategy such as the one proposed in our work and the classical statistical setup of design of experiments is that they provide guarantees on the concentration of parameters conditional on having observed sufficiently many experiments with particular arrangements, but not guarantees on the optimality of the learned model up to that point. Our goal is to learn the most accurate model possible regardless of the number of experiments performed.

Previous work in drug-target prediction has generally addressed active learning methods or drug-target prediction methods, but rarely both. For example, active learning has been used to identify active compounds from a large pool of compounds targeting a single molecule [9]. Active learning has also been applied in the context of cancer research [10]. Several methods for drug-target prediction without active learning have been proposed recently [11–17] and remain an active area of research. The focus of this work is not to promote a particular drug-target prediction method, but to show using matrix factorization as an example of how drug-target prediction can be combined with active learning and lead to reductions of experimentation cost. Initial results on applying active learning for drug-target prediction on multiple drugs and multiple targets simultaneously have been reported [18, 19], with and without requiring prior knowledge of drug or target similarities. Dramatic benefits of active learning on a large dataset from PubChem using drug and target similarities have been reported, but without consideration of when to stop experimentation [19]. A method for predicting the accuracy of models learning by active learning for the purpose of developing a stopping rule has been described, but it was not applied to the particular problem of drug-target prediction [18].

Several stopping rules for active learning have been considered in the past [20–22], however there has been little analysis of which performs the best in general. Four simple stopping criteria based on confidence estimation over the unlabeled data pool and the label consistency between neighboring training rounds of active learning have been presented [22]. Instead of using a single criterion to stop, combining different stopping criteria in a feature vector describing the active learning trajectory has been proposed [18]. The features of trajectories on simulated data are used to train a regression function in order to predict the accuracy of active learning algorithms on unseen simulated data. Here we will follow this approach and adopt it to the binary drug-target prediction case.

The major goals of our active learning system are: (1) We want to have a fast and reliable method to elucidate drug-target interactions. (2) Previous knowledge on similarities between drugs and similarities between targets should be included in the model, so that predictions for new drugs or targets (for which no experiments are available) are possible. (3) The number of experiments required to make confident predictions should be systematically reduced. (4) An efficient stopping rule for ending the active learning process should be designed.

Previously, kernel-based matrix factorization [23] has been shown to provide good models of drug-target interactions [24]. In the kernelized Bayesian matrix factorization (KBMF) algorithm [24, 25], the drug-target interaction matrix is factorized by projecting the drugs and the targets into a common subspace, where the projected drug matrix and the projected target matrix can be multiplied in order to produce a prediction for the drug-target interaction matrix. The entries of the prediction matrix are modeled using truncated normal distributions. The projected drug matrix and target matrix are based on two different kernels: a drug specific kernel and a target specific kernel. A kernel encodes the similarity between the drug and the target features. Thus prior information can be easily inserted into the model. Furthermore, the knowledge of the full interaction matrix is not needed in order to make predictions for new drugs, which is not the case for previous methods (i.e. [12]).

The main contributions of this work are: (i) We use KBMF to construct a powerful and practical active learning strategy for analyzing drug-target interactions. (ii) We extend previous work [18] on estimating the accuracy of active learning predictions to the KBMF case and show how it can be used to construct a stopping rule for experimentation. (iii) We provide a proof of concept through evaluation of the method on four data sets previously used for modeling of drug-target interactions [26]. (iv) We show the superiority of the proposed active learning approach compared to random choice of an equivalent number of experiments.

## Methods

### Active learning framework

**Fig. 1:**
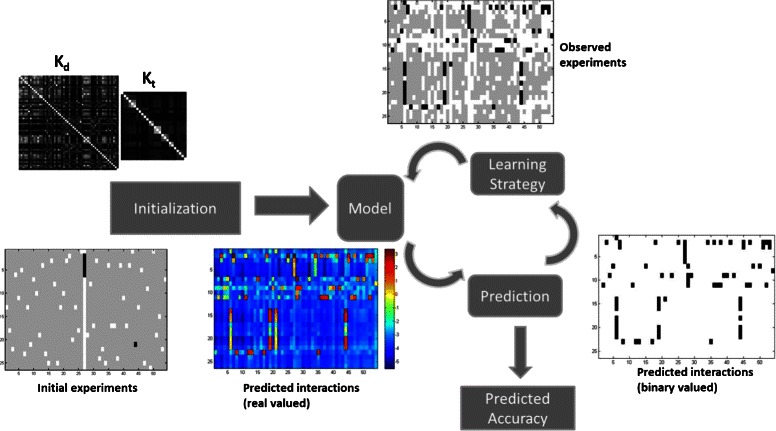
The major components of an active learning framework. The entries of the matrix are color coded: label not known (light gray), interaction (black), no interaction (white). At initialization a subset of known labels for the interactions matrix and the drug and target kernels *K*_*d*_ and *K*_*t*_ are provided. In each round of the active learning algorithm, the labels of the entire interaction matrix are predicted and used to determine which labels to query next. In this figure, the dark red values represent a high probability for a hit, whereas the dark blue values represent a high probability for a miss

An active learning method is an iterative process composed of four components: the initialization, the model, the active learning strategy and an accuracy measure for the predicted output in each step (Fig. 1). Most active learning papers focus on the second and third components. The active learning framework starts with an initialization strategy which is followed by the generation of a model. The model is used to make predictions, in our application drug-target interactions are predicted. Interactions can be measured by performing an *experiment*, i.e. a direct assay of drug-target interaction (e.g., in cell extracts). Based on the predictions, an active learning strategy is applied to query new experiments (labels) which will improve the model. We use batchwise learning, where a fixed number of experiments is queried in each training round thereby increasing the size of experiments with known label. Each training round defines a *time-point* in the active learning process and is measured by the number of batches of experiments performed. For each time-point the accuracy of the model is predicted. The process is stopped for example, if a certain budget for performing experiments is reached or the predicted accuracy of the model is high enough. We assume equal cost for each experiment.

### Data representation

We use interaction matrices **Y**∈{−1,1}^*N*×*M*^ to represent drug-target interactions. We assume that the outcome of the experiment determines the ground truth label $l \in \mathcal L = \{-1,1\}$ for an interaction matrix entry. $N \in \mathbb {N}$ is the number of drugs, $M \in \mathbb {N}$ is the number of targets. Knowledge of the interaction between a drug *d*∈{1,2,...,*N*} and a target *t*∈{1,2,...,*M*} is ternary encoded in the *experimental matrix* **X**: +1 for an interaction, −1 for lack of interaction, and 0 to denote experiments which have not yet been performed. Hereby, the set of remaining experiments (unlabeled data) will be denoted by $\mathcal {X} = \{x = (d,t)|\mathbf {X}(x) = 0 \}$. Therefore, we consider a semi-supervised binary labeling problem where the sign of the label indicates the interaction status between a drug and a target.

### Kernelized Bayesian matrix factorization (KBMF)

We use drug and target kernel matrices respectively to represent the pairwise similarity of drugs to one another and the pairwise similarity of targets to one another. These similarities are values between zero and one, where zero indicates no similarity and one indicates the highest similarity. All the values on the diagonal of the kernel are therefore one. In order to compute the similarities for the target kernel matrix we use the normalized Smith-Waterman score [27] which uses the sequence information of two proteins to compute similarities. Other possibilities to compute the similarity between proteins are to first compute features using programs like ProtParam [28] or Prosite [29] as employed previously [19] and then compute the similarity between the features using a distance metric. For computing the similarity between drugs we used SIMCOMP [30], a program which uses graphs to represent drugs and computes the similarity between two drugs by searching the maximal common subgraph isomorphism. Other tools to compute the similarity between drugs are included in the OpenBabel package [31].

As described previously [24, 25], KBMF can be effectively applied to model drug-target interactions. It approximates the interaction matrix by projecting the drug kernel $\mathbf {K_{d}}\in \mathbb {R}^{N \times N}$ and the target kernel $\mathbf {K_{t}}\in \mathbb {R}^{M \times M}$ into a common subspace of dimension $R \in \mathbb {N}$ such that the interaction matrix **Y** can be reconstructed from the sign of its prediction matrix $\mathbf {F} \in \mathbb {R}^{M \times N}$:

(1) $$ \hat{\mathbf{Y}}(d,t) = \left\{ \begin{array}{rl} 1 & \quad \text{if}\,\, \mathbf{F}(d,t)>0\\ -1 & \quad \text{else}. \end{array} \right.  $$

The prediction matrix **F** is a product of the projected kernel matrices:

(2) $$ \mathbf{F} = \left((\mathbf{A_{d}})^{T}\mathbf{K_{d}}\right)^{T}\left((\mathbf{A_{t}})^{T}\mathbf{K_{t}}\right),  $$

where $\mathbf {A_{d}}\in \mathbb {R}^{N \times R}$ and $\mathbf {A_{t}}\in \mathbb {R}^{M \times R}$ are subspace transformation matrices computed by the variational Bayes algorithm [24, 25] using the values of the experimental matrix **X**. The dimension *R* of the subspace is a free parameter; we used the value of 20 previously determined to be optimal for these datasets [25]. The entries of the kernel matrix *K*_*d*_ and *K*_*t*_ are a measure of the pairwise similarities between drugs and targets respectively. The similarity matrices provided by Yamanishi et al. [26] and the KBMF implementation of semi-supervised classification provided by Goenen [25, 32] were used.

Note that it is not possible to factor the interaction matrix **Y** by multiplying the drug and target kernels directly, since they are matrices of differing dimension. Therefore transformation matrices *A*_*d*_ and *A*_*t*_ are needed which project the drug kernel and the target kernel into a common subspace. Since the product of the transformed kernels **F** should reflect the observed experiments as well as possible, the values of *A*_*t*_ and *A*_*d*_ are found such that they maximize the posterior probability of having observed the experimental matrix **X** along with some prior information on the distribution of the elements in the transformation matrices. Goenen [24, 25] used a graphical model to represent the relationships, and provided a detailed derivation of an efficient inference scheme using variational approximation. The KBMF algorithm is an iterative algorithm which converges usually after 200 iterations. The values of the kernels do not necessarily have to be in the range zero to one, since the scaling of the kernels is implicitly encoded in the transformation matrices.

### Initialization and experiment selection

Our initialization strategy is to select a random column and one random experiment from each row of the experimental matrix **X**.

#### Uncertainty sampling

We use uncertainty sampling [33] to form a batch of experiments $\{x_{1},..,x_{K}\}\in \mathcal {X}$ by greedily choosing the $K\in \mathbb {N}$ experiments with the greatest uncertainty function *U* [22]:

(3) $$ U(x)=-\sum\limits_{l \in \mathcal{L}} P(l|x)log P(l|x),  $$

where $\mathcal {L} = \{0,1\}$ is the set of possible labels and *l* is a label.

For the KBMF case the posterior probability is computed by the sigmoid function from the predicted interactions:

(4) $$ P(l=1|x) = \frac{1}{1+\exp(-\mathbf{F}(x))},  $$

and *P*(*l*=−1|*x*)=1−*P*(*l*=1|*x*) for no interaction respectively.

Here we make use of the property of the KBMF method, that the magnitude of the predicted entry in **F** is an indicator for the confidence of the prediction.

### Stopping rule

In order to stop the active learning process, a method is needed to predict the accuracy of the model for a given time-point along with the confidence of that prediction. As proposed previously in [18], the accuracy of a model at a given point in an active learning process can be predicted using a regression function trained for other, similar experimental spaces. The fully observed drug-target space is characterized by two measures, uniqueness (*u*) and responsiveness (*r*) [18] defined by:

(5) $$\begin{array}{@{}rcl@{}} r &=& \frac{1}{N\cdot M}\sum\limits_{d,t,Y(d,t)=1}\mathbf{Y}(d,t) \end{array} $$

(6) $$\begin{array}{@{}rcl@{}} u &=& \frac{uRows(\mathbf{Y})+ uColumns(\mathbf{Y})}{N+M}, \end{array} $$

where *u* *R* *o* *w* *s*(.) and *u* *C* *o* *l* *u* *m* *n* *s*(.) compute the number of unique rows and unique columns of a matrix.

The uniqueness and responsiveness are values in the range [0,1] and characterize the interaction matrix. Responsiveness measures the percentage of interactions in the matrix. Uniqueness is a measure of independence of the rows and columns in the matrix. The higher the value for uniqueness is, the more difficult it is to make predictions.

These two measures have two purposes: (1) They are used to compute features for a time-step in our current active learning process. (2) They can be used to generate simulation data having similar properties to the measured experimental data.

Each time-point *t*_*i*_ is described by a vector of 13 features $f_{t_{i}} \in \mathbb {R}^{p}$, *p*=13, defined as:

- *f*(1),*f*(2): average observed responsiveness across columns (respectively rows)
- *f*(3),*f*(4): average predicted responsiveness across columns (respectively rows)
- *f*(5): average difference in predictions from last prediction for current time-point (*t*_*i*_)
- *f*(6): average difference in predictions from last prediction for previous time-point (*t*_*i*−1_)
- *f*(7): fraction of predictions at *t*_*i*−1_ observed as responsive (*l*=1) at *t*_*i*_
- *f*(8),*f*(9),*f*(10): minimum, maximum and mean number of experiments that have been performed for any drug
- *f*(11),*f*(12),*f*(13): minimum, maximum and mean number of experiments that have been performed for any target

These features are normalized to the range [0..1] and additional features are generated by computing the square root of their pairwise products (a simple way to create quadratic terms in the regression models). The extended feature vector $\widetilde {f}$ is formed by concatenating the entries $\sqrt {f(i)*f(j)}$, *i*,*j*∈{1,2,..,*p*} and *i*>*j* to the original feature vector. These extended feature vectors $\widetilde {f}$ are predictor variables $\mathbf {f}^{i} =(\tilde {f}_{i1},...,\tilde {f}_{i\tilde {p}})^{T}$, while the true accuracies are stored as rows in the vector of observations $\mathbf {y} \in \mathbb {R}^{N_{f}}$. Therefore, our predictor follows a linear model:

(7) $$\begin{array}{@{}rcl@{}} {}\!\hat{\beta} \,=\,\arg \!\min\!\!\left\{\!\!\sum\limits_{\!i\,=\,1}^{\!N_{f}} \!\left(\!y_{\!i}-\!\!\sum\limits_{\!j\,=\,1}^{\!\tilde {\!p}}\!\beta_{j}\tilde{f}_{i1}\!\right)^{\!2}\!\!\right\} \quad \!\text{\!subject to}\, \!\sum\limits_{j=1}^{\!\tilde{\!p}} |\beta_{j}| \!\leq\! t\! \end{array} $$

where *N*_*f*_ is the number of observations used, $\tilde {p}=0.5\cdot p \cdot (p+1)$ and *t*≥0 is a tuning parameter. We use lasso regression [34] to learn the vector of response coefficients $\mathbf {\beta } \in \mathbb {R}^{\tilde {p}}$.

To learn the accuracy predictor via simulation data, interaction matrices of size 50×50 were randomly sampled in the grid of uniqueness and responsiveness parameters 5 *%*,10 *%*,…,95 *%*. For each interaction matrix we derived ’perfect’ Gaussian similarity kernels *K*_*d*_,*K*_*t*_ by pairwise distances of the column-space and row-space, respectively. These were disrupted by forcing 0 *%*,5 *%*,10 *%* of the kernel entries to the value 1 and regularized to ensure positive semidefiniteness. Features computed from trajectories of the uncertainty sampling active learner on these data were collected; for eachtrajectory we also measured the accuracy of prediction against the ground truth. A linear model of these features against adjusted accuracies (accuracy above the fraction of experiments performed so far) was fitted by lasso regression [34]. The lasso regularization parameter was chosen by 11-fold cross validation under squared loss, with holdout granularity at the level of trajectories. To make accuracy predictions from adjusted accuracy predictions, we added the fraction of experiments performed so far.

## Results

For evaluation of our method, experiments were performed on four data sets extracted from the KEGG BRITE [35], BRENDA [36], SuperTarget [37] and DrugBank [38] databases, previously described by Yamanishi et al. [26, 39]. The data set consists of four drug-target interaction matrices: Nuclear Receptor, GPCR, Ion Channel and Enzyme. To do this evaluation, we considered what a fair comparison would be with how a multiple drug, multiple target screening process might be carried out using current practice. Most decisions about experimental choice are currently made by investigators based on their prior knowledge. However, we are not aware of any study where human investigators have been asked to choose experiments in a multiple drug, multiple target scenario. This would be difficult since investigators would typically not have sufficient knowledge of hundreds of targets to carry this out; most investigators have expertise for a limited number of targets. The closest strategy used in practice would be to choose drugs for each target independently, either using multiple experts or using a strategy such as Quantitative Structure-Activity Relationship (QSAR) modeling [40]. We have previously shown that an AL based strategy outperforms a single-based target strategy panel of experts using QSAR [19]. Therefore, we decided to compare our strategy with random sampling, since random selection is often difficult to improve upon [41].

### Comparison of active and random learning strategies

**Fig. 2:**
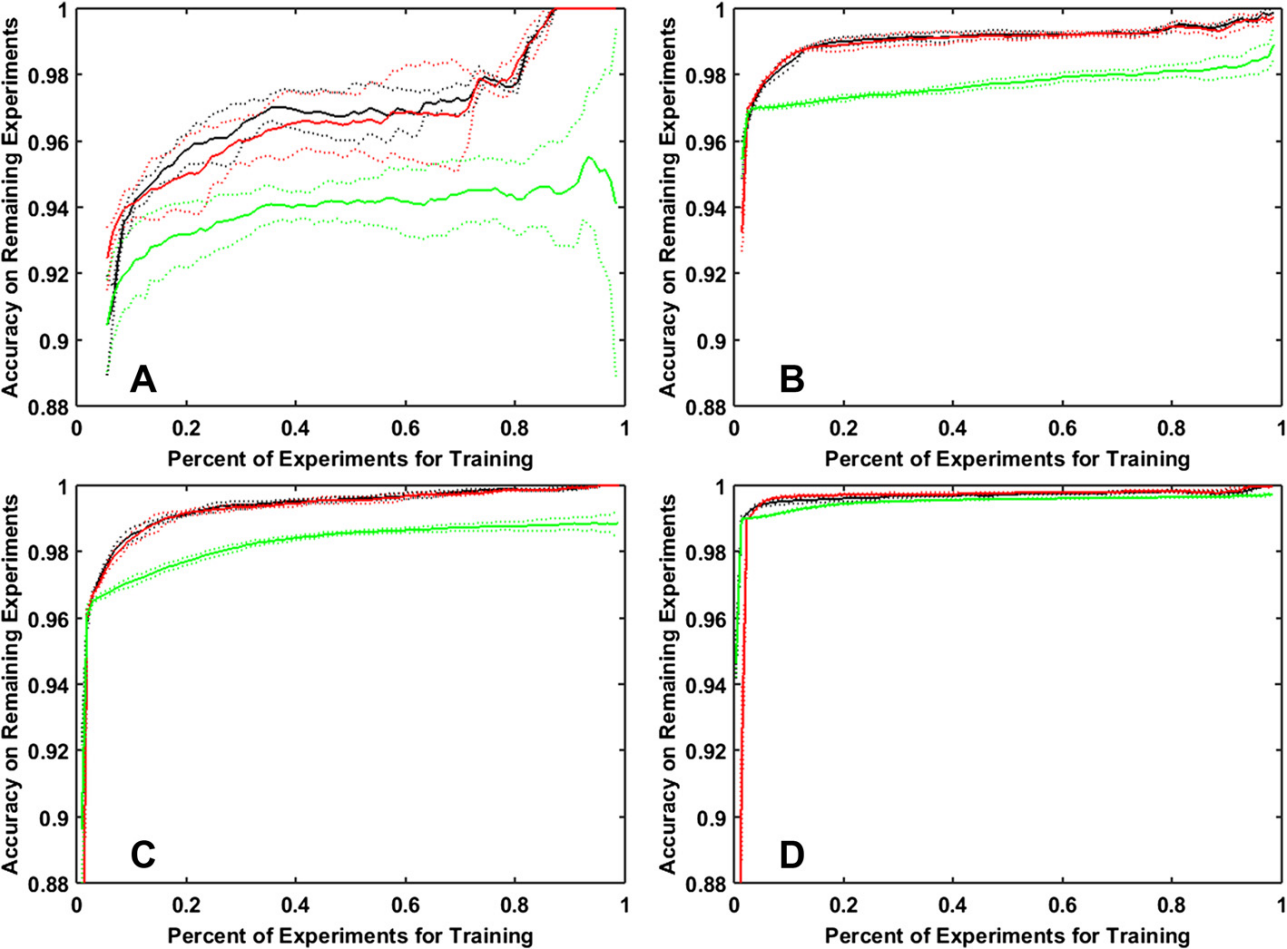
Comparison of random sampling (green) to uncertainty sampling (black: random column initialization, red: diversity based initialization) on the four data sets: (**a**) Nuclear Receptor, (**b**) GPCR, (**c**) Ion Channel, (**d**) Enzyme. The solid and the dotted line represent respectively the mean and the standard deviation of 5 random initializations. For random sampling five random runs were performed for each initialization

As can be seen in Fig. 2, the active learning strategy outperformed the random sampling strategy for all four datasets. The experimentation cycle for both random sampling and uncertainty based sampling was initialized by the same set of experiments, however in each consecutive experimentation cycle the labels that are added to the training set depended on the sampling strategy used (means and standard deviations are shown for 5 different randomly-chosen starting sets). In both cases the KBMF method is used to train the model using the known labels of experiments and make predictions for the labels of the remaining experiments (the accuracy reported is only for these remaining experiments). Thus the accuracies for the first 1 % are the same but they quickly diverge after that. In the very last step of the process 99 % of the experiments are used for training and the prediction performance is evaluated only on the remaining 1 % of the experiments (since this remaining set differs between random and uncertainty based sampling, we can arrive at different final prediction accuracies). On the GPCR and the Ion Channel datasets, the active learning strategy reaches 99 % accuracy 5-6 times faster than the random strategy. We have also tested a second initialization strategy for active learning, where the drugs were clustered and the targets were clustered using k-means clustering. The initialization was performed using the same number of experiments from each drug-target cluster combination. Figure 2 shows that the two initialization strategies for uncertainty sampling yielded similar results.

### Predicting the accuracy of the model

**Fig. 3:**
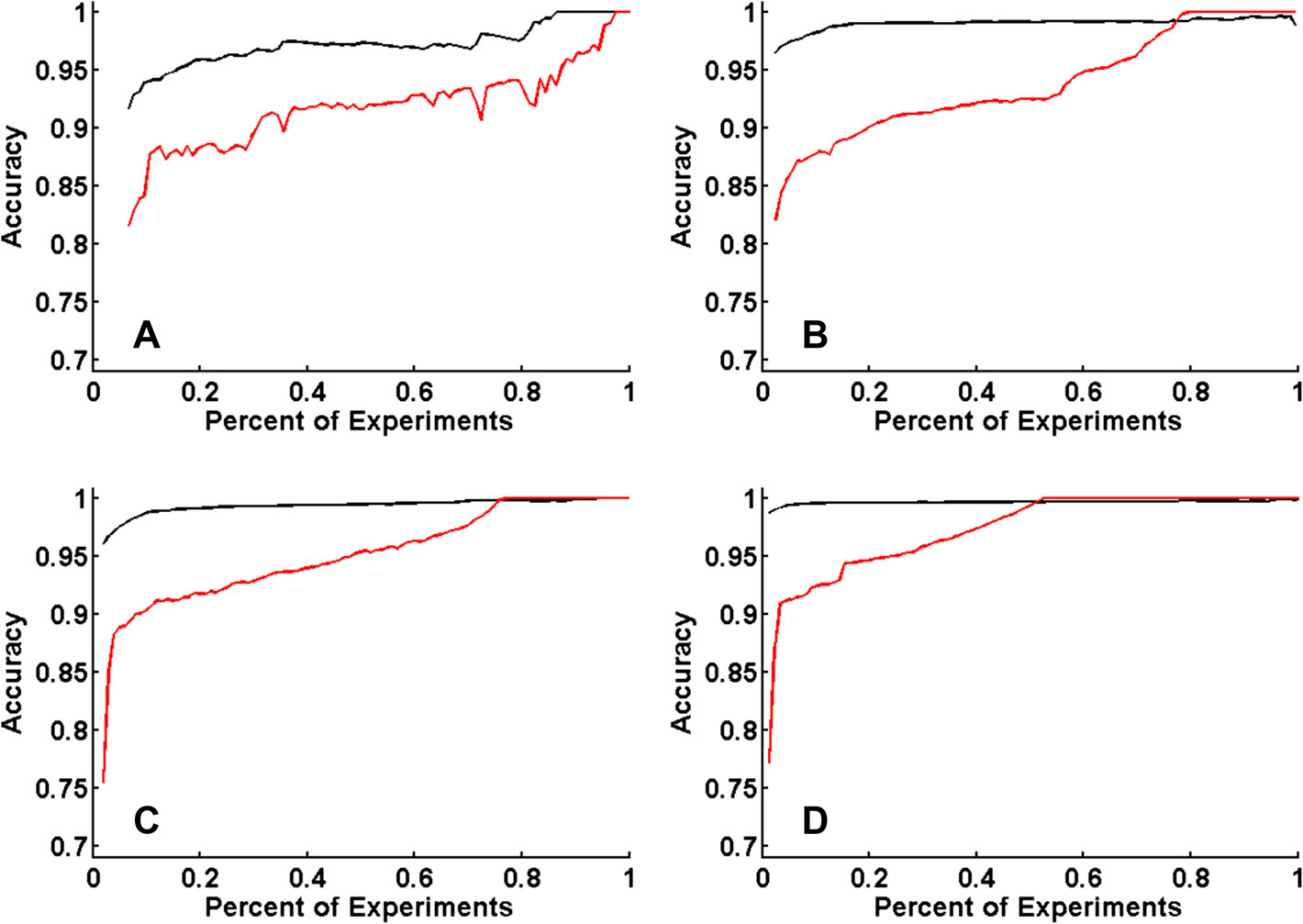
The true accuracy (black) and the predicted accuracy (red) are shown for the four data sets: (**a**) Nuclear Receptor, (**b**) GPCR, (**c**) Ion Channel, (**d**) Enzyme

As discussed above, in practice we require a mechanism to decide when to stop experimentation. It is not enough to have a good active learning method without the possibility to evaluate the accuracy of the whole model apart from acquiring all the data. To address this problem, we have previously proposed a parametrization of perturbagen-target systems in which we characterize each system by its *responsiveness* (the probability that a perturbagen has an effect on a target) and its *uniqueness* (the probability that a perturbagen or target is different from others) [18]. This permits simulations of large numbers of systems to evaluate active learning strategies. We applied this approach by creating many simulated systems for interaction matrices with uniqueness and responsiveness values in the range 0.05−0.95 and with kernel noise in the range 0−0.1. We then performed active learning simulations using our KBMF model and uncertainty sampling and learned a regression function for the predicted accuracy. By uniformly varying the parameters of uniqueness and responsiveness in the range 0.05-0.95, a wide range of possible interaction matrices are generated without the limitation to a special case (a subset of possible interaction matrices). From the interaction matrices the ground truth similarity matrices can be computed by considering the similarity between the rows (target kernel) and the similarity between columns (drug kernel). The ’perfect’ similarity kernels are then disrupted by noise in order to deal with more realistic similarity matrices. It is true that the performance can be improved much further by considering only a subset in the parameter space, however in general it is not known beforehand what parameters describe the considered interaction matrix. Therefore the learned model describes a large range of possible interaction matrices. The results of applying the regression function to the computed features at each time point are shown in red in Fig. 3 for the four experimental data sets. On all four data sets, the predicted accuracy of 90 *%* guarantees the true accuracy to be at least 90 *%*, and the predicted accuracies are a reasonable lower estimate for the true accuracy. Note that a predicted accuracy of 100 %, does not imply that the true accuracy is 100 %. It is merely a prediction from the features at that time point applying the learned regression model and therefore does not indicate that the system has been overfit.

### Learning the stopping rule

**Fig. 4:**
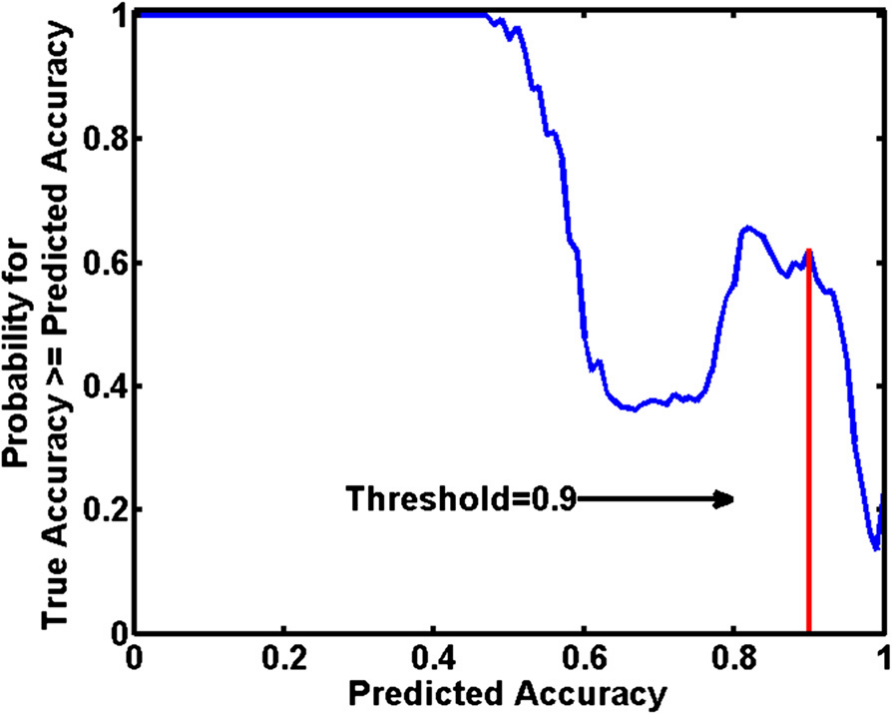
The probability that a predicted accuracy is below or equal to the true accuracy is plotted against the threshold

Statistics on the performance of the accuracy predictor in simulations can be used to design a stopping rule [18]. We adopt this method to determine a threshold for stopping the active learning procedure. The simulated data is used to assess the probability that the true accuracy is greater than or equal to the predicted accuracy using 11-fold cross-validation. The number of folds for cross-validation is essentially an arbitrary choice. By choosing 11-fold cross validation over 10-fold cross validation we have a bit more training data available in each round. We count for each predicted accuracy value how often the condition was fulfilled and divide it by the total occurrence of this predicted value (Fig. 4). As expected, a low predicted accuracy has a high probability that the accuracy measured in the actual experiments will be higher. In the beginning of the active learning procedure a small amount of data is available, so it is hard to make good predictions about the accuracy of the method. However, the more data is gathered in the active learning procedure, the more confident the predictor gets, reaching a peak for predicting the accuracy of 0.8 and higher for 65 *%* of the cases. For very high accuracies (>0.95), the chance that the actual accuracy exceeds the prediction naturally drops drastically. From Fig. 4 the best threshold to stop lies in the range 0.8 to 0.9. Since higher accuracy values are more desirable, our stopping rule was to terminate the active learning procedure when the predicted accuracy was 0.9.

### Applying the stopping rule

In the work of Goenen [24], the KBMF classifier was evaluated by 5-fold five cross validation using 80 *%* of drugs for training and 20 *%* of drugs for testing. We wanted to test if a matching accuracy on the test set could be reached by actively choosing a reduced number of experiments for training. In other words, assuming that we get to perform selected experiments drawn from a given set, use them to train a model, and make predictions for a held out set (for which experiments are not possible), can we get an accurate model without doing all experiments? For this purpose our active learning strategy was modified. We use 1 *%* of drugs as the batch size and select in each run the drugs which the classifier is the most uncertain about. For uncertainty sampling using the predictions of the KBMF classifier, this means that drugs with the maximal mean uncertainty across targets are selected. When the predicted accuracy on the training set reaches 90 %, the active learning process is stopped and the AUC value on the test set (the 20 *%* of the drugs which were held out) is reported. (Note that the stopping rule is to achieve an expected accuracy for the *training* set, and the accuracy for the *test* set would normally be lower.) The average results after 5-fold five cross validation are reported in Table 1. By using the stopping-rule on all four data sets, only half of the drugs were needed for training to reach a similar AUC value to that when using all 80 % of the drugs for training.

**Table 1:** Average AUC on hold out data and percentage of experiments after applying our stopping rule. The average AUC obtained on held out data using 80 *%* of the data for training. Random sampling of the training data [24] is compared with sampling the training data by active learning (AL) and sampling by pre-clustering of the drugs. Furthermore, the average AUC obtained by training with only the listed percentage of experiments obtained by applying the stopping rule is provided. The percentage of experiments can be halved by using the proposed stopping rule

	Goenen results	Pre-clustering	AL	With stopping rule	
Dataset	AUC (*%*)	AUC (*%*)	AUC (*%*)	AUC(*%*)	experiments (*%*)
NR	82.4	84.0	93.6	81.7	52.9
GPCR	85.7	86.4	90.6	81.6	39.3
IC	79.9	85.3	86.8	83.8	44.2
Enz	83.2	85.8	90.3	77.8	29.7

We also tested whether simply clustering drugs according to their similarity could lead to a better training set (similar to the strategy of identifying a ’representative set’ of drugs for screening). We applied k-means clustering on the drug similarity matrix and the number of clusters was chosen using the Akaike information criterion. A set of drugs was chosen to maximize representation of this clustering, either of a fixed size of 80 % or of the same size as that found for a particular dataset by active learning. This approach performs slightly better than random choice of drugs but not as well as active learning selection (Table 1).

### Comparison of stopping rules

We also compared two previously described stopping criteria, overall uncertainty (OU) and minimum expected error (MEE) (either with a fixed threshold or an adapted threshold based on label consistency as described [22]), with our stopping method based on predicted accuracy (Table 2). We use the absolute difference of the percentage of experiments completed at the stopping time-point to the percentage of experiments completed at the best stopping time (BST) averaged over four data sets (*Δ*_*ave*_) to evaluate different stopping criteria, as described previously [22]. The BST is defined as the time-point (fraction of experiments), when the classifier first reaches the highest performance. The predicted accuracy (PA) method with threshold 0.9 produces the smallest average error to the BST. Both MEE and OU perform two to three times worse than the PA method, even with the adaptive threshold method. The fixed threshold for OU and MEE fails on average, because each of the four data sets has a different optimal threshold for OU and MEE. The maximum uncertainty (MU) and the selected accuracy (SA) stopping criteria [22] could not be applied, since those curves are not continuous on these data sets.

**Table 2:** Average difference between the BST point and the stopping point chosen by various stopping rules. OU=Overall Average Uncertainty, MEE=Minimum Expected Error PA=Predicted Accuracy. The value in the parentheses denotes the threshold. The smaller the difference *Δ*_*ave*_ value is, the better the stopping criterion is. The proposed method (PA) with threshold 0.9 performed the best

Methods	OU(0.12)	OU(0.09)	OU(0.06)	OU(0.03)	OU(adapted)
*Δ*_*ave*_(%)	40.1 (± 12.2)	33.8 (± 17.8)	40.1 (± 21.3)	50.9 (± 5.4)	28.2 (± 29.1)
Methods	MEE(0.12)	MEE(0.09)	MEE(0.06)	MEE(0.03)	MEE(adapted)
*Δ*_*ave*_(%)	40.1 (± 11.7)	38.3 (± 12.7)	36.1 (± 13.4)	40.6 (± 12.1)	30.3 (± 12.6)
Methods	PA(0.85)	PA(0.9)	PA(0.95)		
*Δ*_*ave*_(%)	32.8 (± 8.8)	**13.7** (± 11.3)	22.1 (± 15.4)		

## Discussion and conclusions

We have presented an active learning method for prediction of drug-target interactions based on kernelized matrix factorization. Building on prior work [24], our model can efficiently leverage prior information through kernels to achieve high predictive accuracy. We have furthermore shown that our method can significantly improve the prediction task for drug-target interactions when only a limited number of experiments can be performed. For three real-world data sets with high uniqueness values, the active learning strategy achieves 99 *%* accuracy with 2-3 times fewer experiments than a random sampling strategy. It is important to note that our goal was not to choose the best possible matrix completion method for these specific datasets, but to show that a good method can be used as a basis for active learning to dramatically reduce further experimentation.

It should therefore be emphasized that the presented framework is not limited to KBMF only. Any other model for drug target prediction could be applied that produces outputs for drug-target scores which can be converted into probabilities. Furthermore the selection strategy we used (uncertainty sampling) could be replaced by any other active learning strategy (i.e. diversity sampling) to learn new traces on simulated data. Presumably, the regression model for predicting accuracy from simulated active learning traces could also be improved.

For a practitioner to realize these advantages, we have provided a method for estimating the accuracy of an actively learned model using only experimental results already collected; this estimated accuracy is generally a lower bound of the true accuracy of the model. We have shown that this method, calibrated from simulation data, accurately assesses the active learner performance on our real-world data. We have also shown that by applying a stopping rule learned on the simulated data, only half of the experiments are needed to achieve similar accuracies on holdout data. We conclude that active learning driven experimentation is a practical solution to large experimental problems in which time or expense make exhaustive experimentation undesirable.
